# Fermented *Dendrobium officinale* polysaccharides protect UVA‐induced photoaging of human skin fibroblasts

**DOI:** 10.1002/fsn3.2763

**Published:** 2022-02-07

**Authors:** Yongtao Zhang, Shiquan You, Dongdong Wang, Dan Zhao, Jiachan Zhang, Quan An, Meng Li, Changtao Wang

**Affiliations:** ^1^ 58276 Beijing Advanced Innovation Center for Food Nutrition and Human Health Beijing Technology and Business University Beijing China; ^2^ Chemistry and Materials Engineering Beijing Technology & Business University Beijing China; ^3^ Institute of Cosmetic Regulatory Science Beijing Technology and Business University Beijing China; ^4^ Yunnan Baiyao Group Co., Ltd. Kunming China

**Keywords:** antiaging, antioxidative, *Dendrobium officinale* polysaccharides, fermentation, Nrf2, TGF‐β

## Abstract

In this study, Fourier transform infrared spectroscopy (FT‐IR), gel permeation chromatograph‐liquid chromatography (GPC‐LC), and scanning electron microscopy (*SEM*) were used to analyze the molecular characteristics of fermented *Dendrobium officinale* polysaccharides (FDOP) by *Lactobacillus delbrueckii bulgaricus*. The characteristic structural peak of FDOP was more prominent, showing a smaller molecular structure, and its porous structure showed better water solubility. The protective effect of FDOP on the damage of human skin fibroblasts (HSF) caused by ultraviolet (UV) radiation was investigated by evaluating its antioxidative and antiaging indices. The results showed that the antioxidant capacity of HSF was improved, and the breakdown of collagen, elastin, and hyaluronic acid was reduced, thus providing effective protection to the skin tissue. The antioxidative property of FDOP was explored using Nf‐E2‐related factor 2‐small interfering RNA‐3 (Nrf2‐siRNA‐3) (Nrf2‐si3) and qRT‐PCR (quantitative reverse transcription polymerase chain reaction), and the antiaging property of FDOP was explored using Western Blot and qRT‐PCR. The results show that FDOP can up‐regulate signal transduction of the Nrf2/Keap1 (Kelch‐like ECH‐associated protein 1) and transforming growth factor‐β (TGF‐β)/Smads pathways to reduce antioxidative damage and antiaging effects. Therefore, this study provides a theoretical basis for FDOP as a novel functional agent that can be used in the cosmetic industry.

## Graphical abstract

The regulation of human skin fibroblast (HSF) protection mechanism explored by fermented *Dendrobium officinale* polysaccharides (FDOP) in this experiment is roughly shown in Figure 7. It is through stimulating the conduction of Nf‐E2‐related factor 2/Kelch‐like ECH‐associated protein 1 (Nrf2/Keap1) and transforming growth factor‐β (TGF‐β)/Smads signaling pathways to upregulate the expression of downstream target genes.

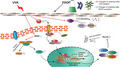

## 1 INTRODUCTION

Skin aging is divided into two types, namely, inevitable endogenous aging and avoidable exogenous aging (photoaging). Photoaging is a phenomenon of cell damage and aging caused by a series of skin oxidative stress reactions caused by long‐term exposure of the skin to ultraviolet (UV) rays (Jadoon et al., 2015). In particular, long‐wave ultraviolet radiation (320–400 nm, UVA) can reach the skin dermis (Favrot et al., 2018), causing oxidative damage, extracellular matrix (ECM) degradation, and melanin hyperplasia in human skin dermal fibroblast cells (Lan et al., 2019; Moreno et al., 2020), mainly manifested as skin wrinkles, dryness, and darkening, which are characteristic manifestations of skin aging. In severe cases, inflammation and skin cancer may occur.

*Dendrobium officinale* (*D. officinale*), known as one of the “Nine Immortals” of Chinese herbal medicine, is a rare medicine found in China. It is widely used in foods, medicines, and cosmetics (Ng et al., 2012), and contains a variety of functional ingredients. Among them, *D. officinale* polysaccharide is a type of heteropolysaccharide, which mainly contains mannose, galacturonic acid, glucose, galactose, rhamnose, arabinose, and others (Ma et al., 2018). Previous studies have shown that it has antitumor, blood pressure lowering, immune regulatory, antioxidative, and antiaging activities (Guo et al., 2020; Guo, Guo, et al., 2020; Liang, Peng, et al., 2018; Liang, Chen, et al., 2018; Lin et al., 2018; Zhao et al., 2019).

In recent years, plant polysaccharides have become a hot topic to be explored for their antioxidant and antiaging active properties. Several studies have indicated that plant polysaccharides improve antioxidant enzyme activity, scavenge free radicals, inhibit lipid peroxidation, protect biofilms, have antiaging properties, and so on (Liu et al., 2018, 2019; Trommer & Neubert, 2005; Wang et al., 2016). Nf‐E2‐related factor 2 (Nrf2) plays an important role in cellular defense against various harmful stimuli, including UVA radiation (Ikehata & Yamamoto, 2018). Plant polysaccharides mainly prevent UV damage by regulating the Nrf2/Keap1 pathway (Liang, Peng, et al., 2018; Liang, Chen, et al., 2018), which is mainly reflected in the obvious improvement of intracellular reactive oxygen species (ROS), lipid peroxidation levels, and antioxidant enzyme levels. According to a previous report (Huang et al., 2020), many natural polysaccharides are too large to enter cells to exert biological effects, or some of their molecular structures show weak activity. However, the use of various microorganisms to convert macromolecular compounds into smaller molecular compounds can improve their biological activity (Guo, Qi, et al., 2020; Guo, Guo, et al., 2020; Huang et al., 2019). In our previous studies (You et al., 2021), we verified changes in the activity of active substances before and after fermentation of the polysaccharides obtained from *Panax notoginseng*. *Lactobacillus delbrueckii bulgaricus* is a widely distributed member of the genus *Lactobacillus* and is one of the bacterial species widely used as probiotics and microbial starters in the food industry (Kok & Hutkins, 2018; Yamamoto et al., 2021).

However, till date, there is no research conducted to explore the protective effects of fermented *Dendrobium officinale* polysaccharides (FDOP) on human skin fibroblasts (HSF) caused by ultraviolet radiation. This study will focus on protective mechanisms on the skin by FDOP from antioxidative and antiaging effects, and analyze the changes in polysaccharides after fermentation using FT‐IR, GPC‐LS, and *SEM*. Nrf2‐small interfering RNA (Nrf2‐siRNA) and qRT‐PCR were used to verify the regulatory effect of FDOP on the Nrf2/Keap1 pathway. The effect of FDOP on the TGF‐β/Smads pathway was also verified by Western Blot and qRT‐PCR to explore antioxidative and antiaging mechanisms of FDOP. This study provides a scientific basis for the application of FDOP as an antioxidant and antiaging agent in foods, medicines, and cosmetics, and expands the current understanding of the synergistic antioxidant effect of Chinese herbal fermented products of natural plant polysaccharides.

## 2 MATERIALS AND METHODS

### 2.1 Materials and chemicals

*Dendrobium officinale* was purchased from Zhejiang Yiwu Xianhe Co., Ltd. *Lactobacillus delbrueckii bulgaricus* (ATCC 11842) was purchased from the Global Bioresource Center. HSF were purchased from the Cell Resource Center of the Institute of Basic Medicine, Chinese Academy of Medical Sciences.

Ascorbic acid (analytical purity ≥99.7%) was purchased from Sinopharm Chemical Reagent Co., Ltd., 0.25% (with EDTA) trypsin was purchased from GIBCO Life Technologies Company. Dulbecco's modified Eagle medium (DMEM), fusion medium (FM), newborn calf serum, phosphate‐buffered saline (PBS), 1 × 10^5^ U/L penicillin and 100 mg/L streptomycin, and Lipofectamine^®^ RNAiMAX Reagent were purchased from GIBCO Life Technologies Company. Trans‐minimal essential medium (Trans‐MEM) reduced‐serum medium was purchased from Guangzhou Yufeng Biotechnology Co., Ltd. EasyScript^®^ one‐step genomic DNA (gDNA) removal and complementary DNA (cDNA) synthesis SuperMix reverse transcription kit (TransStart^®^ Top Green qPCR SuperMix kit) were purchased from Beijing Quanshijin Biotechnology Co., Ltd. Total glutathione peroxidase (GSH‐px) assay kit with nicotinamide adenine dinucleotide phosphate (NADPH), lipid peroxidation malondialdehyde (MDA) assay kit, catalase assay kit, reactive oxygen species (ROS) detection kit, radioimmunoprecipitation assay (RIPA) lysis buffer, and Cell Counting Kit 8 were purchased from Biorigin (Beijing) Inc. Hyaluronan enzyme‐linked assay (ELISA) kit, human elastase ELISA kit, human collagen Type I ELISA kit, human collagen Type III ELISA kit, human total matrix metalloproteinase 1 (MMP‐1) ELISA kit, human total matrix metalloproteinase 3 (MMP‐3) ELISA kit, and human NRF2 ELISA kit were procured from Cusabio Biotech Co., Ltd.

### 2.2 Microbial cultivation and fermentation

*Lactobacillus delbrueckii bulgaricus* was activated, and the activated bacterial strain was inoculated into de Man, Rogosa and Sharpe (MRS) medium and cultured in an oscillator (Qilinbeier, China) at 37°C.

*Dendrobium officinale* was dried and passed through a 50‐mesh sieve to obtain a powder, which was then sterilized at high temperature with a solid‐to‐liquid ratio of 1:15 (*D. officinale* powder: water), inoculated with 5% bacterial culture, and allowed to stand for 48 h in a constant temperature incubator at 37°C. Water, instead of 5% bacterial culture, was used as a control for the fermentation extraction method.

### 2.3 Crude polysaccharide extraction

Each extract was centrifuged at 4°C at 4800 rpm (RJ‐LD‐50G, Ruijiang Analysis Instrument Co., Ltd.) for 20 min, and the supernatant thus obtained was mixed with absolute ethanol at a volume ratio of 1:4, and the sample was precipitated overnight at 4°C. After alcohol precipitation, it was redissolved in water to the original volume of the sample, 2% papain was added (v/v) and mixed well. Next, enzymolysis was carried out at 25°C for 4 h, and the sample was boiled for 10 min to inactivate the enzyme; later, alcohol precipitation was performed again (Huang et al., 2021). The collected sediments were freeze‐dried to form crude polysaccharides of *D. officinale*, which were designated as fermented *D. officinale* polysaccharides (FDOP) and water‐extracted *D. officinale* polysaccharides (DOP).

### 2.4 FT‐IR analysis

Concentrations of 1.0 mg/ml of FDOP and DOP were prepared, and the characteristic functional groups on the sugar chains of FDOP and DOP were analyzed by infrared spectroscopy. The range of the wavelength used was 600–4000 cm^−1^, and the resolution was 4 cm^−1^.

### 2.5 Morphology and molecular weight analysis

According to the method of Yang et al. (Yang et al., 2018), the FDOP sample was fixed on *SEM* stubs and covered with a thin (10 nm) layer of gold (Au) before observation by *SEM* (S‐4800, Hitachi). The surface micromorphology of the sample was observed using *SEM* with an acceleration voltage of 15.0 kV at 300× magnification.

Molecular weight was determined using gel permeation chromatography‐light scattering (GPC‐LS) using a series of columns, such as Ultrahydrogel 250 Column (7.0 × 300 mm), Ultrahydrogel 120 Column (7.0 × 300 mm), and Ultrahydrogel Guard Column 125 (6 µm, 6 mm ×40 mm). The FDOP and DOP samples were prepared as 3 mg/ml solutions (the injection volume was 20 µl), and 0.2 M sodium nitrate (containing 0.02% sodium diazide) was used as the mobile phase (0.8 ml/min; Det: 50°C; Col: 60°C) to elute FDOP and DOP.

### 2.6 Measurement of antioxidant activity in vitro

*Dendrobium officinale* polysaccharides and FDOP samples were diluted with deionized water in four gradients as test solutions, and 2,2‐diphenylpicrylhydrazyl (DPPH) free‐radical scavenging activities of the two test solutions were measured according to the method of Gupta et al. (Gupta et al., 2015). After a reaction time of 30 min, absorbance was measured at 517 nm. The Fenton method of Luo et al. (Luo et al., 2009) was referenced to evaluate the scavenging rate of DOP and FDOP on hydroxyl radicals (HO); the absorbance was measured at 536 nm after the reaction. The pyrogallol method of Zhao et al. (Zhao et al., 2007) was referenced to evaluate the scavenging rate of DOP and FDOP on superoxide anion radicals (·O_2_^‐^). In our study, the absorbance was measured at 320 nm using a microplate reader (BioTek Instruments). Sample group (T), sample bottom value group (T0), blank group (C), and solvent bottom value group (C0) were all set in the experiments. The free radical scavenging rate was calculated according to the following formula:

$$\text{Scavenging effect(\%)}=1‐\left(T‐T0\right)/\left(C‐C0\right)\times 100\%$$

### 2.7 Establishment of cell culture and UVA‐damage model

Human skin fibroblasts were cultured in DMEM supplemented with 10% fetal bovine serum (FBS) and 1% penicillin‐streptomycin. The cells were incubated in a 37°C Heracell CO_2_ incubator (Thermo Fisher Scientific Co., Ltd.) at 5% CO_2_, and the medium was changed every 2 days. All tests were performed on cells obtained between 4th and 6th passages.

Next, 100 μl of the HSF cell suspension (8 × 10^4^–1 × 10^5^ cells/ml) was added to each well of a 96‐well plate. After 12 h, the culture medium was replaced with the DOP and FDOP in sample wells and UVA control wells, respectively, and the plates were irradiated at 5 mW/cm2 under a UVA lamp (Spectronics Corporation, Ltd) for 2 h. The cells were then incubated in a constant temperature and humidity incubator at 37°C and 5% CO_2_ for 24 h.

### 2.8 Assay of cell viability

Next, 100 μl of HSF cell suspension (8 × 10^4^–1 × 10^5^ cells/ml) was added to each well of a 96‐well plate, preincubated for 12 h, and then the medium was changed. Five concentrations of sample solution were added to the sample wells, serum‐free DMEM was added to the control wells and blank wells, and the plate was incubated for 24 h; next, 10 μl of CCK‐8 was added to each well and incubation was carried out further for 2 h. Absorbance was measured at 450 nm and the cell viability was calculated to observe the toxicity of FDOP and DOP.

After 24 h of incubation, the established UVA‐damaged cell models (sample group and UVA control group) were added to basal medium DMEM in a 96‐well plate, 10 μl of CCK‐8 was added to each well and incubated for 2 h. The absorbance was measured at 450 nm and the cell viability was measured to observe the protective effect of FDOP and DOP on HSF.

### 2.9 Measurement of total antioxidant capacity

Human skin fibroblasts were collected, the cells were lysed and centrifuged at 12,000 rpm (Allegra X‐30R, Beckman Coulter, Inc.) for 10 min at 4°C, and the supernatant was collected. The experiment was carried out using the total antioxidant capacity test kit (ABTS (2,2'‐azino‐bis(3‐ethylbenzothiazoline‐6‐sulfonic acid) method) according to the manufacturer's instructions. OD_734_ was measured using a microplate reader, and the total antioxidant capacity of the sample was calculated according to the standard curve.

### 2.10 Measurement of intracellular ROS levels

Intracellular ROS content was determined using ROS detection kit according to the manufacturer's instructions. The cells were added to a 6‐well plate, inoculated with 2 ml/well of 2'‐7'dichlorofluorescin diacetate (DCFH‐DA) (10 μM) in the established cells, and incubated for 20 min. A fluorescence microplate reader (Tecan M200 Infinite Pro) was used to measure the fluorescence intensity of the samples at 495 nm excitation and 545 nm emission wavelengths.

### 2.11 Measurement of antioxidant enzyme

According to the protocol mentioned in the catalase (CAT) detection kit, 4 μl of the prepared hydrogen peroxide solution and 200 μl of the color‐developing working solution were added into the 96‐well plate, incubated at 25°C for at least 15 min, and a microplate reader was used to determine OD_520_ and draw a standard curve. Next, a 4‐μl cell lysate was added to a 1.5‐ml test tube, and 37 μl of catalase detection buffer and 10 μl of 250 mM hydrogen peroxide were added. The contents were mixed well and incubated for 5 min, after which 450 μl of catalase reaction termination solution was added to terminate the reaction. Next, 40 μl of catalase detection buffer was added to 10 μl of the above‐mentioned reaction mixture, incubated at 25°C for 15 min, and then OD_520_ was measured using a microplate reader. Catalase activity was calculated according to the given formula.

Detection buffer, the HSF cell lysate to be tested, and GPx detection working solution were added sequentially to a 96‐well plate and mixed well. Next, the plate was incubated at room temperature for 15 min, and 10 μl of 30 mM peroxide reagent solution was added to each well and the contents mixed well. An appropriate microplate reader or micro‐ultraviolet spectrophotometer was immediately used to measure A_340_ at 25°C, and the absorbance was measured every one min, for a total of six sets of data. Glutathione peroxidase (GSH‐px) enzyme activity was calculated according to the formula mentioned in the kit.

### 2.12 Lipid peroxidation assay

As much as 0.1 ml of PBS was added to a test tube as a blank control, 0.1 ml of different standard concentrations was added to the above calibration curve tube; the HSF cell lysate (0.1 ml) was then added, followed by the addition of 0.2 ml malondialdehyde (MDA) detection fluid. After mixing, the mixture was heated in a boiling water bath for 15 min, cooled to room temperature in a water bath, and centrifuged at 1000×*g* for 10 min. The supernatant (200 μl) thus obtained was added to the 96‐well plates and the absorbance was measured using a microplate reader at 532 nm. The MDA content was calculated according to the formula mentioned in the kit.

### 2.13 ELISA

After the cell culture supernatant was collected, the HSF were irradiated for 24 h as per the ELISA kit instructions, and MMP‐1, MMP‐3, transforming growth factor‐β (TGF‐β), Smads, collagen type I (COL‐I), collagen type III (COL‐III), elastin (ELN), Nrf‐2, Keap1, heme oxygenase 1 (HO‐1), NAD(P)H:quinone oxidoreductase 1 (NQO‐1), and hyaluronan were analyzed by ELISA.

### 2.14 Transfection

Human skin fibroblasts (8 × 10^4^–1 × 10^5^ cells/well) were inoculated into a 6‐well plate and cultured in DMEM without antibiotics until the confluence rate reached 60%–80%. Nine microliters of Lipofectamine^®^ RNAiMAX Reagent and 3 µl (10 µM) Nrf2‐siRNA at a ratio of 1:1 were added to 150 µl of Trans‐MEM^®^ and then allowed to stand at room temperature for 15 min. A 250 µl of the siRNA–lipid mixture was added to each well containing HSF and incubated for 2 days. The sequence information of Nrf2‐siRNAs is shown in Table S1.

### 2.15 Western blot

Cellular proteins were extracted and analyzed by Western Blot. A 6%–12% sodium dodecyl sulfate‐polyacrylamide gel was first used to isolate the proteins, which were then transferred to a nitrocellulose membrane (GE Whatman). The membranes were blocked with 3% bovine serum albumin (BSA) for 1 h and then incubated with appropriate primary and secondary antibodies. Chemiluminescent images were obtained using an ImageQuant LAS 4000 Mini (GE Healthcare Life Sciences) (Yeh & Yen, 2006).

### 2.16 qRT‐PCR

Total RNA was extracted and detected by agarose gel electrophoresis (Chen et al., 2019; Wu et al., 2019). According to the gene sequence obtained from the National Center for Biotechnology Information (NCBI), Primer Express software was used to design specific primers for the housekeeping gene β‐actin, as shown in Table S2. EasyScript^®^ one‐step gDNA removal and cDNA synthesis SuperMix reverse transcription kit was used for cDNA first‐strand synthesis reaction; TransStart^®^ Top Green qPCR SuperMix kit and real‐time quantitative reverse transcription PCR (ABI7300, Thermo Fisher Scientific Co., Ltd.) were used for the experiments.

### 2.17 Statistical analysis

Three separate experiments were conducted for all experiments, and each sample underwent three technical repeats and analyses. Data are expressed as mean ±standard deviation. One‐way analysis of variance (ANOVA) and Dunnett's test were used to analyze the data to determine which pairs were significantly different from each other. Statistical analyses were conducted using GraphPad Prism 9 (GraphPad Software, Inc.). A value of *p* < .05 was considered to be statistically significant.

## 3 RESULTS

### 3.1 Characterization of molecular structures of FDOP and DOP

**FIGURE 1:**
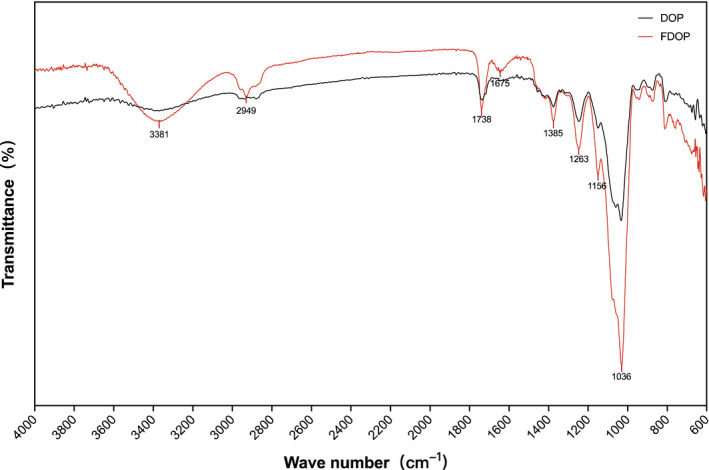
FT‐IR spectra of water‐extracted *Dendrobium officinale* polysaccharides (DOP) and fermented *D. officinale* polysaccharides (FDOP)

As shown in Figure 1, the peak at 3381 cm^‐1^ is due to the stretching vibration of the carbohydrate hydroxyl group (O‐H), while the stretching vibration of methylene‐CH2 appears at 2949 cm^‐1^, the peak at 1738 cm‐1 indicates the stretching vibration of C = O, and the peak at 1385 cm^‐1^ indicates the bending vibration of the C‐H bond. In addition, at around 1036 cm^‐1^, a stretching vibration of the C‐O bond of the carbohydrate compound is observed. Notably, the FT‐IR spectrum of FDOP exhibits higher peaks, especially the stretching vibration peaks at 3381 cm^‐1^ and 1036 cm^‐1^, which are significantly different from those of DOP. This result can be explained by the fact that the glycosidic bonds of the macromolecular polysaccharides of *D. officinale* are broken down into small polysaccharides by enzymes secreted by *Lactobacillus delbrueckii bulgaricus*.

### 3.2 Characterization of microscopic morphology of FDOP

The molecular weights (MW) of FDOP and DOP are listed in Table 1. The MW of FDOP and DOP detected by the laser detector were 1.581 × 10^6^ (±0.950%) and 2.187 × 10^6^ (±1.288%), respectively. It can be seen that the MW of the polysaccharides of *Dendrobium officinale* decreased after fermentation, as shown in Table 1.

**TABLE 1:** Distribution of molecular weights of crude fermented *Dendrobium officinale* polysaccharides (FDOP) and water‐extracted *D. officinale* polysaccharides (DOP)

Peak name	FDOP	DOP
Peak limits (min)	13.575–21.180	13.344–21.004
Mw	1.581 × 10^6^ (±0.950%)	2.187 × 10^6^ (±1.288%)
Mz	5.219 × 10^6^ (±2.340%)	9.107 × 10^6^ (±4.250%)
Mw/Mn	1.607 (±1.262%)	1.536 (±1.575%)
Mz/Mn	5.304 (±2.483%)	6.396 (±4.346%)

**FIGURE 2:**
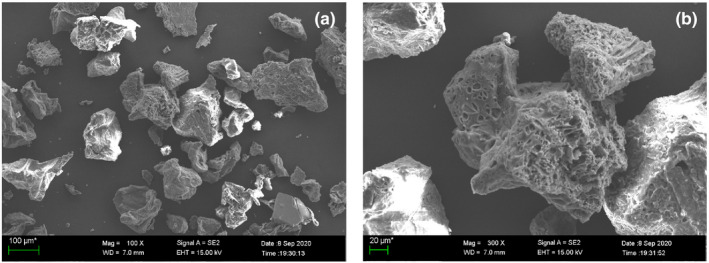
Microscopic morphology of fermented *Dendrobium officinale* polysaccharides (FDOP) under a scanning electron microscope (*SEM*)

When observed under 100× and 300× magnifications of the scanning electron microscope, the surface ultramicroscopic morphological structure of FDOP presented porous foam blocks with irregularities, as shown in Figure 2. This signifies that the texture of FDOP is relatively soft and delicate, which is in good agreement with the appearance of its lightweight powder. FDOP also exhibited stronger water solubility than DOP.

### 3.3 Effects of DOP and FDOP on scavenging free radicals

**FIGURE 3:**
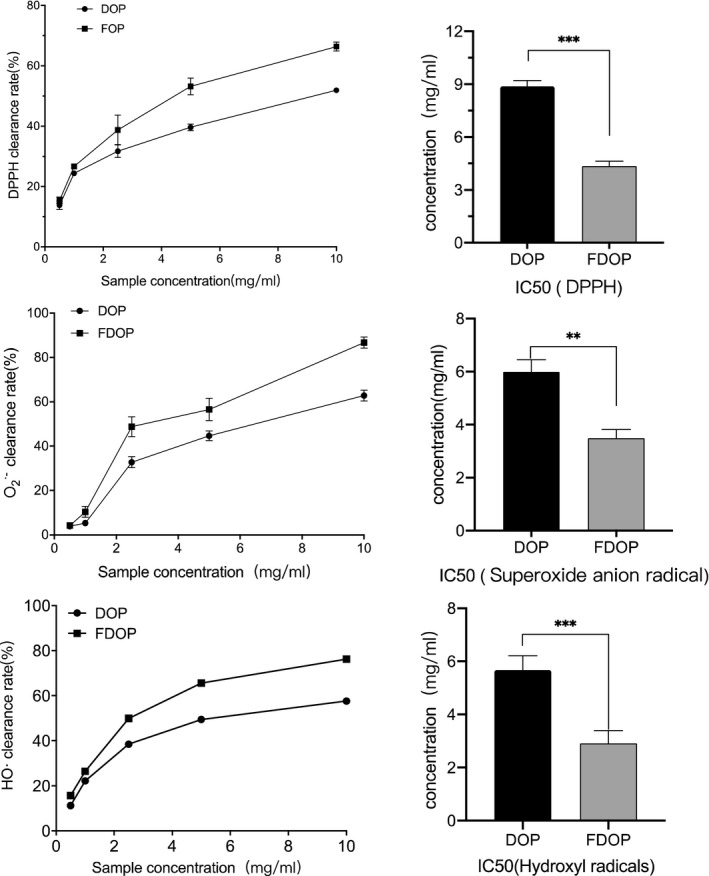
Through in vitro scavenging experiment of 2,2‐diphenylpicrylhydrazyl (DPPH) free radicals (a), superoxide anion free radicals (O2·‐) (b), and hydroxyl radicals (·OH) (c), the free radical scavenging activities of DOP and FDOP at different concentrations (0.5–10.0 mg/ml) were evaluated. Each value is expressed as mean ±standard deviation. Three parallel experiments were performed per group. The difference analysis between DOP and FDOP is indicated by “*” (*: *p* < .05, **: *p* < .01, ***: *p* < .001)

In the in vitro antioxidant experiments, DPPH free radical, superoxide anion free radical, and hydroxyl free radical experiments were used to preliminarily explore the antioxidant activity of FDOP and DOP. Through the analysis of free radical scavenging rate and half scavenging rate (IC50) (Figure 3), the IC50 values of FDOP against DPPH free radicals, superoxide anion free radicals, and hydroxyl free radicals were 4.353 ± 0.276, 3.489 ± 0.327, and 2.91 ± 0.477 mg/ml, respectively, which were about half of the IC50 obtained in the three free radical scavenging experiments of DOP. Preliminary results showed that at the same concentration level, the antioxidant activity of the polysaccharides extracted from *D. officinale* after fermentation was approximately twice that of the unfermented polysaccharides, and there was a significant difference between the two (*p* < .01).

### 3.4 Effects of DOP and FDOP on HSF activity

**FIGURE 4:**
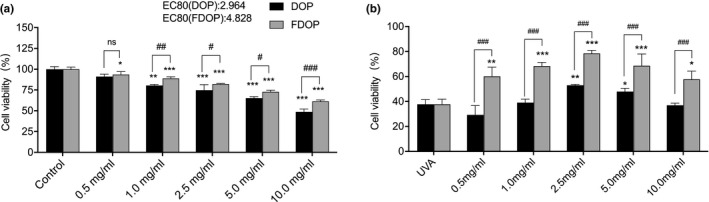
Effects of water‐extracted *Dendrobium officinal*e polysaccharides (DOP) and fermented *D. officinale* polysaccharides (FDOP) on the activity of human skin fibroblasts (HSF) (a) and on ultraviolet radiation (UVA)‐damaged HSF (b). Each value is expressed as mean ±standard deviation. Six parallel experiments were performed per group. The difference analysis between other experimental groups and the UVA model group is indicated by “*” (*: *p* < .05, **: *p* < .01, ***: *p* < .001). The difference analysis between DOP and FDOP is indicated by “#” (#: *p* < .05, ##: *p* < .01, ###: *p* < .001)

A cytotoxicity test was performed to determine the toxic effect of FDOP and DOP on HSF, damage of UVA to HSF, and protective effect of FDOP and DOP on the UVA damage of cells. As shown in Figure 4a, the toxicity of FDOP and DOP to the cells increased in a dose‐dependent manner. The concentrations displaying 80% of the maximal effect (EC80) of FDOP and DOP were 4.828 and 2.964 mg/ml, respectively. The EC80 of FDOP was 1.63 times that of DOP. Taken together, the results suggest that the toxic effect of the polysaccharides extracted from *Dendrobium officinale* after fermentation on HSF was significantly reduced.

As shown in Figure 4b, the survival rate of HSF after UVA‐induced injury under normal culture conditions was as low as 37.69%. The survival rate of injured cells cultured in FDOP (in the range of 0.5‒2.5 mg/ml) increased in a dose‐dependent manner. However, the DOP‐treated group showed a lower survival rate. Statistical analysis showed that there was a highly significant difference between the FDOP and DOP groups (*p* < .001). These results suggest that FDOP has a stronger effect on protecting HSF from UVA damage than DOP.

From the results obtained from the cytotoxicity test, we selected three concentrations, 0.5, 1.0, and 2.5 mg/ml, to perform the subsequent experiments.

### 3.5 FDOP and DOP protect HSF from UVA‐induced oxidative stress

Prolonged exposure to UVA light stimulates cells to produce excessive reactive oxygen species (ROS), thereby stimulating the cells to produce lipid peroxide, which ultimately leads to changes in cell structure and function (Xian et al., 2019). A cell has a mature defense mechanism against the pro‐oxidation effect of ultraviolet radiation, including antioxidant enzymes such as catalase (CAT) and glutathione peroxidase (GSH‐px), as well as the oxidative stress regulation pathway Nrf2/Keap1, which protects skin cells from ROS‐mediated damage (Gȩgotek et al., 2017; Tonelli et al., 2018).

### 3.6 Total antioxidant capacity and active oxygen detection

**FIGURE 5:**
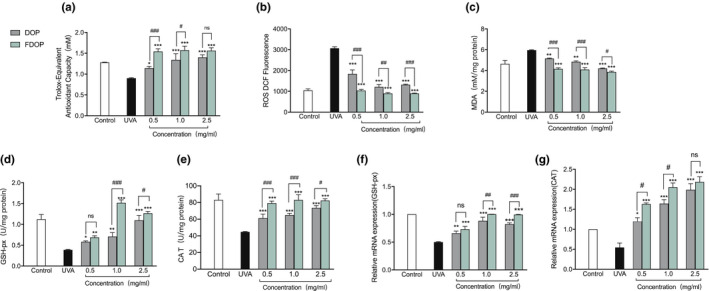
Total antioxidant capacity of fermented *Dendrobium officinale* polysaccharides (FDOP) and water‐extracted *D. officinale* polysaccharides (DOP) (a); effects of FDOP and DOP on reactive oxygen species (ROS) (b); effects of FDOP and DOP on lipid peroxidation (c); effect of FDOP on glutathione peroxidase (GSH‐px) activity (d) and transcription expression (f); and effect of FDOP on catalase (CAT) activity (e) and transcription expression (g). Each value is expressed as mean ±standard deviation. Three parallel experiments were performed in each group. The difference analysis between other experimental groups and the ultraviolet radiation (UVA) model group is indicated by “*” (*: *p* < .05, **: *p* < .01, ***: *p* < .001). The difference analysis between DOP and FDOP is indicated by “#” (#: *p* < .05, ##: *p* < .01, ###: *p* < .001)

In the experiment to determine the total antioxidant capacity of FDOP and DOP, as shown in Figure 5a, the ability of the UVA‐damaged HSF lysate to scavenge ABTS free radicals decreased from 1.28 ± 0.007 mM to 0.9 ± 0.013 mM. In contrast, after incubating with FDOP and DOP, the ability of the HSF lysate to scavenge ABTS free radicals after exposure to UVA was improved. FDOP exhibited better antioxidant activity, and the total antioxidant capacity of 1.0 mg/ml was reached at 1.57 ± 0.1 mM of FDOP.

An increase in ROS content is the most important cause of oxidative damage in cells. In the test to determine the effects of FDOP and DOP on the ROS content in UVA‐damaged HSF, as shown in Figure 5b, UVA caused a significant increase in the ROS level in HSF. Compared to those of the UVA injury model group, the ROS levels of the FDOP and DOP groups were reduced to varying degrees. The effect of FDOP on ROS was dose‐dependent, and the ROS level of the FDOP group was significantly lower than that of the DOP group (*p* < .01). After HSF were treated with DOP and exposed to UVA, the ROS content in the cells was lower than that of direct exposure to UVA. However, after HSF were treated with FDOP and then exposed to UVA, the measured ROS content in the cells was lower than that in the blank control group.

### 3.7 The effects of FDOP and DOP on lipid peroxidation and antioxidant enzymes

The oxygen free radical reaction and lipid peroxidation reaction play important roles in the metabolism of the body. An increase in free radicals can cause lipid peroxidation, which can significantly damage the cell membrane and mitochondrial function of cells and induce cell death (Kong et al., 2020). As shown in Figure 5c, the lipid peroxidation detection experiment suggests that the content of lipid peroxidation product MDA in UVA‐damaged HSF increased significantly. Compared with that of the UVA control group, the intracellular MDA content of the FDOP and DOP groups decreased in a dose‐dependent manner. The effect of FDOP was significantly higher than that of DOP (*p* < .01).

The expression of antioxidant enzymes is mainly regulated by the Nrf2/Keap1 pathway. In an experiment to detect the activity and relative expression of antioxidant enzymes in cells, the activity and relative expression of catalase (CAT) in HSF were decreased twofold after damage caused by exposure to UVA (Figure 5e,g). In addition, as shown in Figure 5d,f, the enzyme activities and expression levels of GSH‐px were significantly decreased. In addition, the activity and relative expression of antioxidant enzymes in cells at some sample concentrations were higher than those in the blank control group; for example, at 1.0 mg/ml FDOP, the CAT enzyme activity was 83.02 ± 6.33 U/mg, the enzyme activity and relative expression of GSH‐px were 1.51 ± 0.075 U/mg and 1.0017 ± 0.001 U/mg, respectively, and the relative expression of CAT at the three concentrations was higher than that of the blank control. These results suggest that FDOP and DOP have significant effects on protecting HSF cells from UVA‐induced damage and play a role in “oxidative stress defense system,”, especially FDOP that plays a stronger role than DOP.

### 3.8 Regulation of Nrf2/Keap1 signaling pathway

**FIGURE 6:**
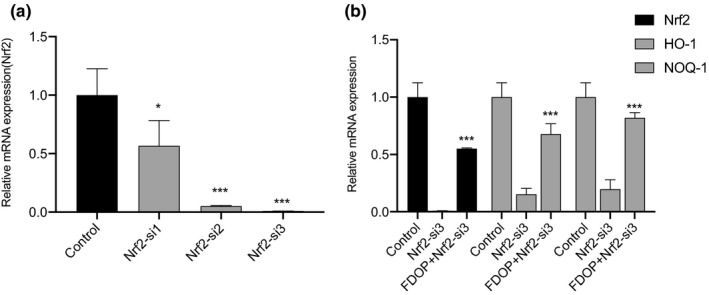
Influence of fermented *Dendrobium officinale* polysaccharides (FDOP) on the Nf‐E2‐related factor 2 (Nrf2) signal pathway of human skin fibroblasts‐Nf‐E2‐related factor 2‐small interfering RNA‐3 (HSF‐Nrf2‐si3). Effects of three siRNAs, Nrf2‐si1/si2/si3, showing the interference of Nrf2 mRNA expression in HSF (a); effect of FDOP (1 mg/ml) on messenger RNA (mRNA) expression levels of Nrf2, hemeoxygenase 1 (HO‐1), and NAD(P)H:quinone oxidoreductase 1 (NOQ‐1) in HSF‐Nrf2‐si3 (b). Each value is expressed as mean ±standard deviation. Three parallel experiments were performed in each group. The difference analysis between other experimental groups and the ultraviolet radiation (UVA) model group is indicated by “*” (*: *p* < .05, **: *p* < .01, ***: *p* < .001). The difference analysis between DOP and FDOP is indicated by “#” (#: *p* < .05, ##: *p* < .01, ###: *p* < .001)

Total RNA of HSF cells transfected with Nrf2‐siRNAs was extracted, and the changes in the expression levels of Nrf2 mRNA were detected by qRT‐PCR. As shown in Figure 6a, the mRNA expression of Nrf2 in HSF transfected with Nrf2‐si2 and Nrf2‐si3 decreased significantly, especially after transfection with Nrf2‐si3, and reached the lowest value of 0.0092. In the next experiment, HSF transfected with Nrf2‐si3 were used to explore the regulation of Nrf2 and downstream antioxidant factors. As shown in Figure 6b, the relative expression levels of Nrf2, OH‐1, and NOQ‐1 mRNA in HSF‐Nrf2‐si3 given with 1.0 mg/ml FDOP were all significantly upregulated. These data are sufficient to show that FDOP regulates the Nrf2/Keap1 signaling pathway, which is a good proof to demonstrate the protective mechanism of FDOP on HSF.

**FIGURE 7:**
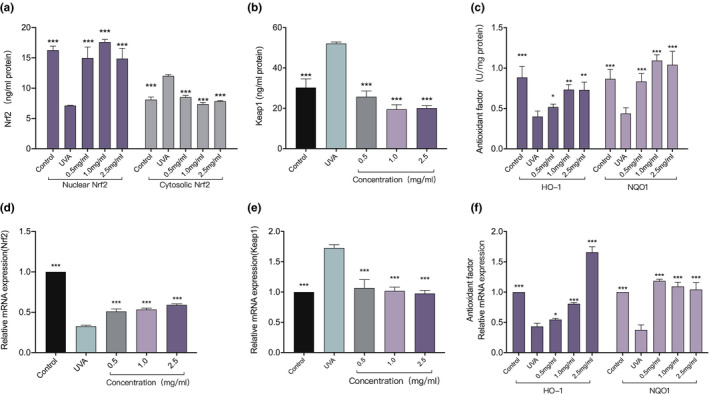
Effects of fermented *Dendrobium officinale* polysaccharides (FDOP) on the Nf‐E2‐related factor 2 (Nrf2) signaling pathway. Effects of FDOP on Nrf2 content (a), transcription expression (d), Kelch‐like ECH‐associated protein 1 (Keap1) content (b), and transcription expression (e). Effects of FDOP on the activities of NAD(P)H:quinone oxidoreductase 1 (NQO‐1) and heme oxygenase 1 (HO‐1) (c) and transcription expression (f). Each value is expressed as mean ±standard deviation. Three parallel experiments were performed in each group. The difference analysis between other experimental groups and the ultraviolet radiation (UVA) model group is indicated by "*" (*: *p* < .05, **: *p* < .01, ***: *p* < .001)

In an experiment to explore the effects of FDOP on the content and expression of Nrf2 and Keap1 proteins in UVA‐damaged HSF cells, Nrf2 protein levels in the nucleus were observed to be reduced by 2.2 times due to abnormal nuclear displacement, so that Nrf2 continued to accumulate in the cytoplasm (Figure 7a). In addition, the relative expression of the Nrf2 gene was decreased by 3 times (Figure 7e). Both the protein content and relative expression of Keap1 were increased by 1.7 times (Figure 7b,f). Compared with the UVA‐exposed control group, the content and relative expression of Nrf2 in the nucleus and cytoplasm of FDOP‐treated HSF cells have extremely significant differences (*p* < .001); in addition, the content and relative expression of the Keap1 protein also showed highly significant differences (*p* < .001). Taken together, these results suggest that UVA stimulation mainly downregulates the antioxidant pathway through nuclear abnormity of Nrf2, downregulation of Nrf2 gene expression, and upregulation of Keap1 gene expression. However, FDPO alleviates the negative effects of UVA on HSF to varying degrees and has obvious protective effects against abnormal nuclear translocation and gene downregulation.

In addition, compared with the UVA model group, the FDOP‐treated HSF exhibited higher relative expression of the downstream target genes of Nrf2/Keap1 and enzymatic activity of transcriptionally expressed antioxidant enzymes HO‐1 and NQO‐1; in particular, FDOP had the most significant effect on NQO‐1, and its enzyme activity and expression were higher than those of the blank control group. These results indicate that FDOP offers varying degrees of protection against UVA damage to HSF.

Combining the data mentioned above, it was observed that the “defense system” of oxidative stress in cells was weakened, mainly due to the downregulation of the Nrf2 pathway, leading to a reduction in the expression of downstream targeted genes and a decrease in the activity of antioxidant enzymes. When HSF were treated with FDOP and then exposed to UVA, the protein content and relative gene expression of Nrf2 and Keap1 in cells and the enzyme activity and relative expression of downstream antioxidant enzymes HO‐1 and NQO‐1 were significantly lower than those directly exposed to UVA. Compared with the blank control group, some concentrations of the samples showed different beneficial effects on these indicators.

### 3.9 Repair effect of FDOP and DOP on the degradation of extracellular matrix

Extracellular matrix (ECM), a structural network that provides firmness and elasticity to the skin, is mainly composed of collagen and elastin (Rousselle et al., 2019). In addition, hyaluronic acid present in ECM acts as a water‐locking agent in the skin. Degradation of ECM results in a decrease in hydration, elasticity, and firmness of the skin (Bonté et al., 2019).

Matrix metalloproteinases (MMPs) and transforming growth factors (TGF‐β) are the main factors that cause ECM imbalance (Choi et al., 2020; Yiu et al., 2008; Yun et al., 2014). For example, MMP‐1 degrades collagen, MMP‐3 degrades elastin, and TGF‐β promotes the expression of ECM genes and inhibits the production of ECM degrading proteins.

#### 3.9.1 Extracellular matrix (ECM)

**FIGURE 8:**
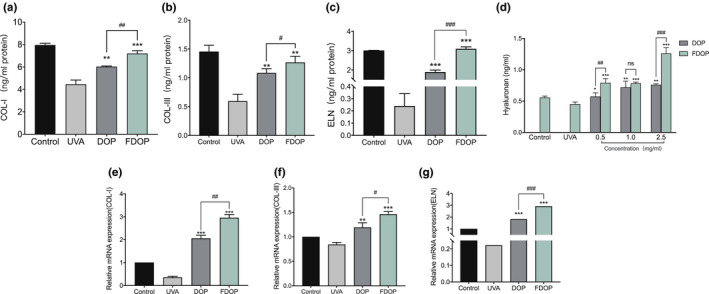
Effect of fermented *Dendrobium officinale* polysaccharides (FDOP) on extracellular (ECM) degradation in ultraviolet radiation (UVA)‐damaged human skin fibroblasts (HSF). Effects of FDOP and water‐extracted *D. officinale* polysaccharides (DOP) on the content of collagen type 1 (COL‐I) (a) and transcription expression (e); effects of FDOP and DOP on the content of collagen type III (COL‐III) (b) and transcription expression (f); effects of FDOP and DOP on the content of elastin (ELN) (c) and transcription expression (g); effects of FDOP and DOP on the content of hyaluronan (d). Each value is expressed as mean ±standard deviation. Three parallel experiments were performed in each group. The difference analysis between other experimental groups and the UVA model group is indicated by “*" (*: *p* < .05, **: *p* < .01, ***: *p* < .001). The difference analysis between DOP and FDOP is indicated by “#” (#: *p* < .05, ##: *p* < .01, ###: *p* < .001)

The experiment determined the effect of FDOP on the extracellular matrix by analyzing the content and expression of the main components of the ECM in the cell cytoplasm before and after stimulation. As shown in Figure 8, compared with the blank group, the content and transcription expression of COL‐I, COL‐III, and ELN in UVA‐damaged HSF decreased to varying degrees. Compared with the HSF in the UVA control group, HSF treated with FDOP and DOP and exposed to UVA displayed significantly lower COL‐I, COL‐III, and ELN content; the gene expression of COL‐I, COL‐III, and ELN in the cells not only increased significantly, but was also higher than that of the blank group.

In addition, compared with that in the blank control, the hyaluronan content measured in the FDOP and DOP groups increased to varying degrees, especially when the FDOP was 2.5 mg/ml. The hyaluronan content increased to 1.26 ± 0.09 ng/ml, this was 2.27 times of the blank group and 1.66 times of DOP group at the same concentration.

#### 3.9.2 Matrix metalloproteinases (MMPs)

**FIGURE 9:**
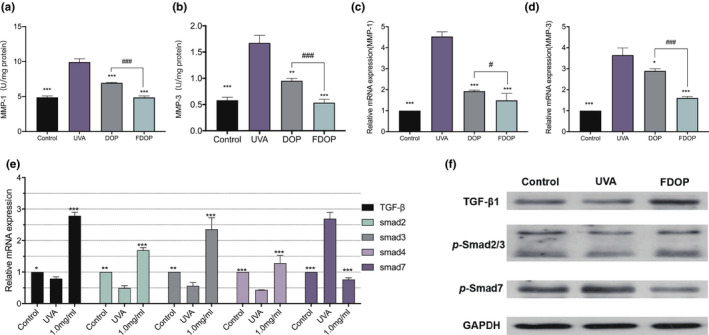
Effect of fermented *Dendrobium officinale* polysaccharides (FDOP) on the activity of matrix metalloproteinase 1 (MMP‐1) (a) and transcription expression (c), effect of FDOP on the activity of matrix metalloproteinase 3 (MMP‐3) (b) and transcription expression (d), and effect of FDOP on transforming growth factor‐β (TGF‐β) and Smads transcription expression (e). The levels of transforming growth factor‐β1 (TGF‐β1), p‐Smad2/3, and Smad7 were detected by Western blot analysis; each protein level was normalized to the ribosomal protein glyceraldehyde 3‐phosphate dehydrogenase (GAPDH) and expressed relative to the control level (f). Each value is expressed as mean ±standard deviation. Three parallel experiments were performed in each group. The difference analysis between other experimental groups and the ultraviolet radiation (UVA) model group is indicated by “*” (*: *p* < .05, **: *p* < .01, ***: *p* < .001)

Matrix metalloproteinases (MMPs) can degrade a variety of extracellular matrices, and the degradation of extracellular matrix causes skin folds and inelasticity (Jabłońska‐Trypuć et al., 2016), which is the main manifestation of skin aging. As shown in Figure 9, in an experiment to study the effects of FDOP and DOP on MMP‐1 and MMP‐3 in UVA‐damaged HSF, compared with the blank group, the enzymatic activity and relative expression of MMP‐1 and MMP‐3 in HSF directly exposed to UVA were significantly reduced (*p* < .001). After HSF were treated with DOP and FDOP, the influence of UVA stimulation was significantly reduced; in particular, the effect of FDOP on MMP‐1 and MMP‐3 in treated cells exceeded or was equivalent to that of normal cells, and the enzymatic activity reached 4.879 ± 0.216 U/mg and 0.535 ± 0.064 U/mg, respectively.

#### 3.9.3 Regulation of TGF‐β/Smads signaling pathway

Transforming growth factor‐β (TGF‐β) and Smad proteins play important roles in regulating cell growth and differentiation. The related pathway proteins of Smads, such as Smad2, Smad3, and Smad4, promote signal transduction of TGF‐β, while Smad7 inhibits it (Luong et al., 2018). As shown in Figure 9, compared with that of the blank group, the expression of TGF‐β and its related pathway proteins (Smad2, Smad3, and Smad4) in UVA‐damaged HSF decreased to varying degrees, while the expression of Smad7 increased significantly. Compared with that in the UVA control group, the relative expression of TGF‐β, Smad2, Smad3, and Smad4, and that of Smad7 showed highly significant differences (*p* < .001), reaching 2.79 ± 0.11, 1.69 ± 0.08, 2.35 ± 0.39, 1.28 ± 0.25, and 0.76 ± 0.057, respectively. These results indicate that UVA can inhibit the expression of TGF‐β in HSF and affect the expression of Smad proteins by affecting signal transduction. However, FDOP can effectively slow down the inhibitory effect of UVA on TGF‐β, thereby reducing UVA damage to skin cells.

Transforming growth factor‐β (TGF‐β) is recognized as a vital mediator in the synthesis of collagen and elastin. To clarify the effects of FDOP on COL‐I and ELN synthesis, the expression of TGF‐β, Smad1, Smad2, Smad3, and Smad7 proteins in HSF exposed to UVA was detected by Western Blot. The results shown in Figure 9f suggest that the protein expression of TGF‐β and Smad2/3 in the FDOP‐treated group significantly increased (*p* < .05) compared with that in the control group, and Smad7 in the FDOP‐treated group decreased dramatically.

## 4 DISCUSSION

*Dendrobium officinale* is a Chinese herbal medicine that has attracted much attention and is used as an important functional component in foods, medicines, and cosmetics. The application of *D. officinale* in cosmetics is limited to water‐extracted mixed components; however, the application of specific or fermentation‐extracted components in skin care products has not been studied till date. In this study, in order to analyze and study the application value of FDOP in skin care, the molecular structure and molecular characteristics of FDOP were analyzed by FT‐IR, GPC‐LS, and *SEM*, and the antioxidative and antiaging effects of FDOP on damaged HSF were explored through the establishment of UVA‐damaged HSF cell models.

**FIGURE 10:**
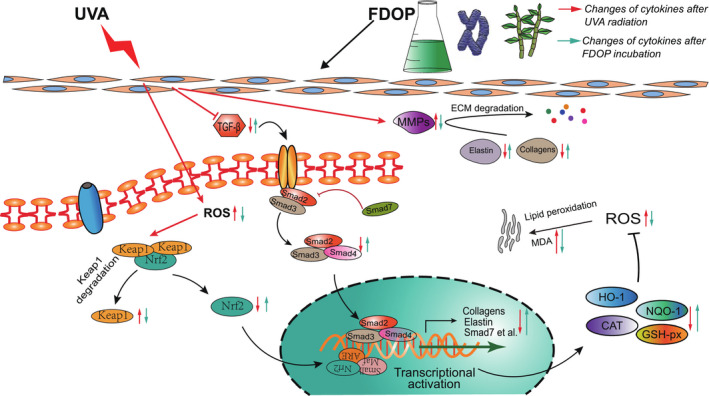
A schematic diagram showing the regulatory mechanisms of the protective effects of *Dendrobium officinale* polysaccharides on ultraviolet (UVA)‐damaged human skin fibroblasts (HSF)

The results of our study suggest that after fermentation of *D. officinale*, the stretching vibration peaks of the hydroxyl O‐H bond of the polysaccharide at 3381 cm^‐1^ and the C‐O bond of the polysaccharide at 1036 cm^‐1^ of FDOP characterized in the FT‐IR spectrum are more prominent than those of DOP. Moreover, the MW of FDOP characterized by GLC‐LS was 1.581 × 10^6^ (±0.950%), which was smaller than that of DOP. This result might be due to the fact that polysaccharides of *D. officinale* were decomposed by *Lactobacillus delbrueckii bulgaricus* into lower molecular weight polysaccharides after fermentation. In addition, the microstructure of FDOP observed by *SEM* shows a porous foam block shape, which is more in line with the characteristics of instantaneous melting of the water present in FDOP. UVA is one of the main causes of the aging of human skin. Our results show that after UVA irradiation of fibroblasts, the levels of ROS and lipid peroxidation in the cells increased, resulting in cell damage. The main proteins of ECM expressed and secreted by cells were also significantly reduced, which reduced the physical support, water retention, and protection of ECM in the cells. This was also accompanied by a significant decrease in the activity of antioxidant enzymes and an increase in the activity of matrix metalloproteinases (MMPs). The effect of FDOP on Nrf2/Keap1 was verified through the use of Nrf2‐siRNAs, and the effect of FDOP on TGF‐β/Smads was verified by Western Blot. The regulation of the protection mechanism of FDOP on HSF is explained as a scheme in Figure 10. It occurs through the stimulation of the Nrf2/Keap1 and TGF‐β/Smads signaling pathways that upregulate the expression of downstream target genes. Our results show that FDOP can enhance the antioxidant enzyme system by regulating the nuclear translocation of Nrf2 and Keap1 to upregulate the Nrf2/Keap1 signaling pathway, and can also reduce ECM degradation by regulating TGF‐β/Smads and MMPs. FDOP show higher antioxidant and antiaging properties. Moreover, our results demonstrate a higher protective effect of FDOP than of DOP, which provided the possibility for a better utilization of *D. officinale* polysaccharides. In our study, the activity of FDOP was significantly higher than that of DOP; its higher activity may be due to the fact that *D. officinale* polysaccharides were decomposed into smaller molecular weight polysaccharides or modified into polysaccharide components with higher activity under the action of *Lactobacillus delbrueckii bulgaricus*.

## CONFLICT OF INTEREST

The authors declare no competing financial interest.

## Supporting information

Tab S1‐S2

## Data Availability

The data that support the findings of this study are available from the corresponding author upon reasonable request.
