# Supplementary material for: Fermented Dendrobium officinale polysaccharides protect UVA‐induced photoaging of human skin fibroblasts
Source: Food Sci Nutr. 2022 Feb 7;10(4):1275–88. doi: 10.1002/fsn3.2763 (PMC9007291; doi:10.1002/fsn3.2763)
Supplement: Supplementary file 1 — Tab S1‐S2 [file FSN3-10-1275-s001.docx]

Effect Of Fermented *Dendrobium Officinal* Polysaccharides Protect UVA-induced Photo-aging In Human Skin Fibroblasts

Yongtao Zhang ^1,2,3^, Shiquan You^1,2,3^, Dan Zhao^1,2,3^, Dongdong Wang^1,2,3^, Jiachan Zhang^1,2,3^, Quan An^4^, Meng Li^1,2,3*^ and Changtao Wang^1,2,3^

^1^ Beijing Advanced Innovation Center for Food Nutrition and Human Health, Beijing Technology and Business University, Beijing, China;

^2^ Chemistry and Materials Engineering, Beijing Technolgoy & Business University, 11 Fucheng Road, Haidian District, Beijing 100048, China;

^3^ Institute of cosmetic regulatory science, Beijing Technology and Business University, Fucheng Road, Beijing 100048, China;

^4^ Yunnan Baiyao Group Co., Ltd., Kunming 650000, China;

* Correspondence: limeng@btbu.edu.cn; Tel.: +86-13426015179

Table S1. Nrf2-siRNAs

Designed and synthesized by Aoke Dingsheng Biological Technology Co., Ltd.

| Primers | Sequence |
| --- | --- |
| Nrf2-si1  sense | 5’-GGUUGAGACUACCAUGGUUTT-3’ |
| Nrf2-si1  antisense | 5’-AACCAUGGUAGUCUCAACCAG-3’ |
| Nrf2-si2  sense | 5’-GCCCAUUGAUGUUUCUGATT-3’ |
| Nrf2-si2  antisense | 5’-AUCAGAAACAUCAAUGGGCCC-3’ |
| Nrf2-si1  sense | 5’-GCAGUUCAAUGAAGCUCAATT-3’ |
| Nrf2-si1  antisense | 5’-UUGAGCUUCAUUGAACUGCTC-3’ |

Table S2. Primers

Designed and synthesized by Aoke Dingsheng Biological Technology Co., Ltd.

| Primers | Sequence | Gene ID |
| --- | --- | --- |
| β-actin | F:5'-TGGCACCCAGCACAATGAA-3’  R:5'-CTAAGTCATAGTCCGCCTAGAAGCA-3’ | 60 |
| CAT | F:5'-CCTTCGACCCAAGCAA-3’  R:5'-CGATGGCGGTGAGTGT-3’ | 847 |
| GSH-px | F:5'-AGAAGTGCGAGGTGAACGGT-3’  R:5'-CCCACCAGGAACTTCTCAAA-3’ | 2976 |
| NQO1 | F: 5’-ATCCTGCGTTTCTGTGGCT-3’  R: 5’-TCCTCCCAGACGGTTTCC-3’ | 1728 |
| HO1 | F:5’-CAAGCGCTATGTTCAGCGAC-3’  R:5’-GCTTGAACTTGGTGGCACTG-3’ | 3162 |
| Nrf2 | F:5’-CAACTCAGCACCTTGTATC-3’  R:5’-TTCTTAGTATCTGGCTTCTT-3’ | 4780 |
| Keap1 | F:5’-GGAGGCGGAGCCCGA-3’  R:5’-GATGCCCTCAATGGACACCA-3’ | 9817 |
| MMP1 | F:5’-TTGAGAAGCCTTCCAACTCTG-3’  R:5’-CCGCAACACGATGTAAGTTGTA-3’ | 4312 |
| MMP3 | F:5’-CCTGATGGCACCCATTTACAC-3’  R:5’-GAGCTCCTGAATGCCCTTGA-3’ | 4314 |
| TGF-β | F:5’-CTGGCGATACCTCAGCAAC-3’  R:5’-TAAGGCGAAAGCCCTCAAT-3’ | 7040 |
| Samd2 | F:5'-ACCGAAAATGCCACGGTAGAA-3'  R:5'-TGGGGGCTCTGCACAAAGAT-3 | 4087 |
| Samd3 | F:5'-GAGGTCTGCGTGAATCCCTA-3'  R:5'-GGAATGGCTAGTCGTCCA-3' | 4088 |
| Samd4 | F:5'-CATCCTGCTCCTGAGTATTGG-3'  R:5'-GGGTCCACGTATCCATCAAC-3' | 4089 |
| Samd7 | F:5'-AGAGGCTGTGTTGCTGTGAA-3'  R:5'-AAATCCATCGGGTATCTGGA-3' | 4092 |
| COL-I | F:5’-CACAGAGGTTTCAGTGGTTTGG-3’  R:5’-GCACCAGTAGCACCATCATTTC-3’ | 1277 |
| COL-III | F:5'-CCTGGTCCTCCTGGTAGT-3'  R:5'-TCCCTTCTCTCCTGGTTG-3' | 1281 |
| ELN | F:5'-GGGTTGTGTCACCAGAAGCA-3'  R:5'-CAACCCCGTAATTAGGAATGC-3' | 2006 |
